# Supplementary material for: Expression of Two α-Type Expansins from Ammopiptanthus nanus in Arabidopsis thaliana Enhance Tolerance to Cold and Drought Stresses
Source: Int J Mol Sci. 2019 Oct 23;20(21):5255. doi: 10.3390/ijms20215255 (PMC6862469; doi:10.3390/ijms20215255)
Supplement: Supplementary file 1 [file ijms-20-05255-s001.pdf]

International Journal of
Molecular Sciences

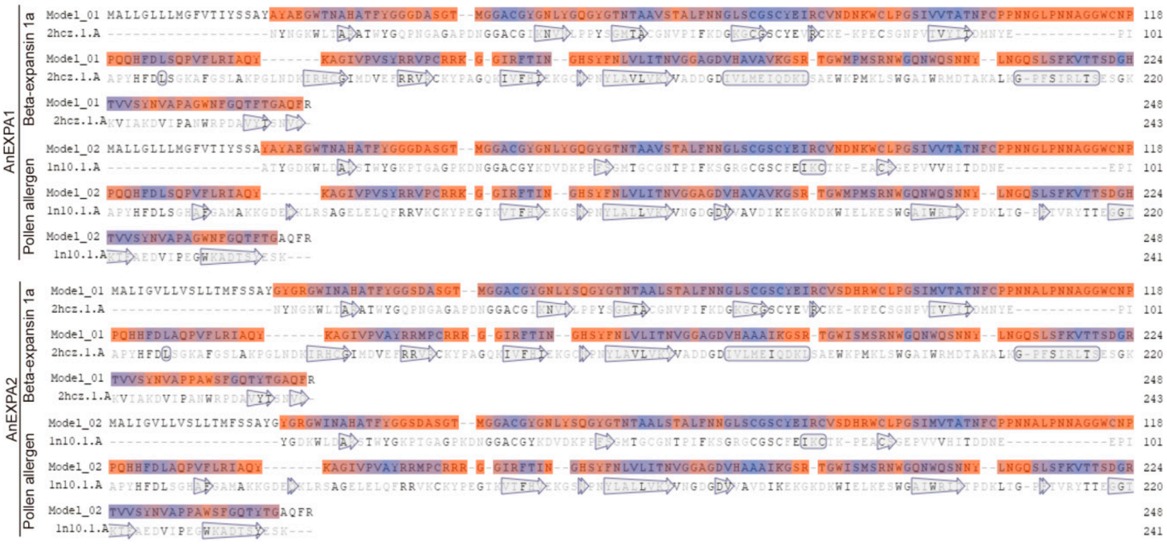

Figure S1. Sequence alignment of the AnEXPA1 and AnEXPA2 proteins with Beta-expansin 1a (2hcz.1.A) and a Pollen allergen (Phlp 1, 1n10.1.A).

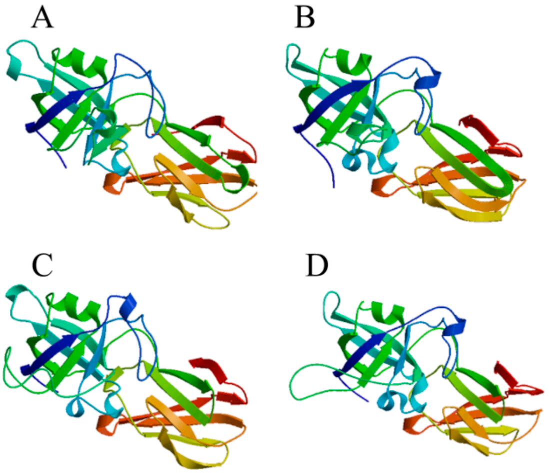

Figure S2. Structural model of AnEXPA1 and AnEXPA2 proteins.

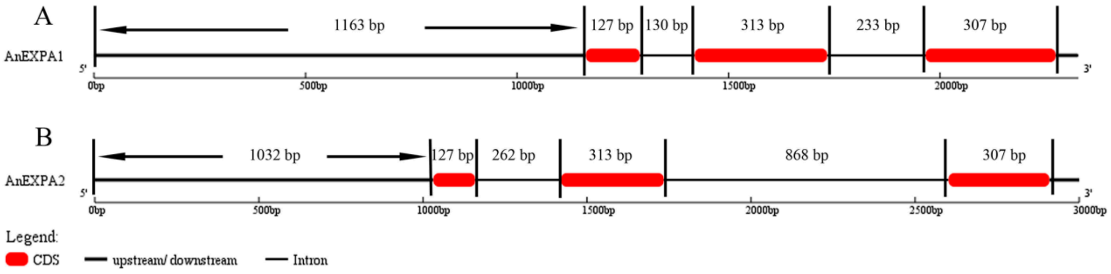

Figure S3. Promoter, exon and intron distribution of *AnEXPA1* and *AnEXPA2*.

Table S1. Sequences of primers.

Primer name	Sequence (5′ -’3)	Description
EXPA1CDS F	TGACCATGGTAGATCATGGCTCTCCTTGGGTTAC	PCR amplification primer, forward; Restriction endonuclease, Bgl II

EXPA1C DS R	ATTTACCCTCAGATCGCGGA ATTGAGCACCAGTG	PCR amplification primer, reverse; Restriction endonuclease, Bgl II
EXPA2C DS F	TGACCATGGTAGATCATGGC TCTCATTGGTGTGCTC	PCR amplification primer, forward; Restriction endonuclease, Bgl II
EXPA2C DS R	ATTTACCCTCAGATCGCGGA ATTGAGCACCAGTG	PCR amplification primer, reverse; Restriction endonuclease, Bgl II
proAnE XPA1 F	CCGAATTCATCACTAGTGCC AAACTAGT	PCR amplification primer, forward; Restriction endonuclease, EcoRI
proAnE XPA1 R	TTCCATGGTTCCTGAACATAT ATACCAAAC	PCR amplification primer, reverse; Restriction endonuclease, NcoI
proAnE XPA2 F	CCGAATTCGTCTGAATCCCTT ACTACTCAG	PCR amplification primer, forward; Restriction endonuclease, EcoRI
proAnE XPA2 R	TTCCATGGTCCTGAACATCCA AAATAAAATAT	PCR amplification primer, reverse; Restriction endonuclease, NcoI
Actin F	ACATTGTCTTGAGTGGTGGTT C	Standard control primer, forward
Actin R	TACTTCCTCTCTGGTGGTGCT A	Standard control primer, reverse
AnEXPA 1 F	AATGGGTGGGGCTTGTGGAT AT	Real-time PCR primer, forward
AnEXPA 1 R	TTTGGTGGGCAGAAGTTAGT GG	Real-time PCR primer, reverse
AnEXPA 2 F	AACCCTCCTCAGCACCACT	Real-time PCR primer, forward
AnEXPA 2 R	TGAACCTTATGCCTCCCCT	Real-time PCR primer, reverse

Table S2. Cis-acting elements in *AnEXPA1* and *AnEXPA2* promoters.

TFs Motif	Sequence	Number		Function of transcription factors (TFs)
		AnEXPA1	AnEXPA2	
MYB1AT	WAACCA	1	3	Dehydration-responsive
MYCCONSENSUSAT	CANNTG	3	1	Regulates the transcription of CBF/DREB1 genes in the cold
ASF-1 binding site	TGACG	1	0	Auxin and/or salicylic acid, abiotic and biotic stress
PYRIMIDINEBOXOSRAMY1A	CCTTTT	3	6	Gibberellin- response cis- element sugar repression
MYBCORE	CNGTTR	1	1	Water stress
GAREAT	TAACAAR	1	0	GA-responsive element
CBFHV	RYCGAC	0	2	Binding site of CBF
CATATGGMSAUR	CATATG	0	1	Auxin responsiveness
MYCATERD1	CATGTG	0	4	Dehydration
RYREPEATBNNAPA	CATGCA	0	2	ABA-induction

MYCATRD22	CACA TG	0	1	Dehydration-responsive; ABA-induction
MYBATRD22	CTAA CCA	0	1	Dehydration-responsive; ABA-induction
WRKY71OS	TGAC	0	2	Gibberellin signaling pathway
